# The relationship between organizational dehumanization and work engagement: the mediating effect of nurses’ work stress

**DOI:** 10.1186/s12912-024-01841-z

**Published:** 2024-03-22

**Authors:** Mennat-Allah G. Abou Zeid, Mahmoud Abdelwahab Khedr, Heba Nasser Rayan, Boshra mostafa, Ayman Mohamed El-Ashry

**Affiliations:** 1 Nursing Administration Department, Faculty of Nursing, Ain Shams University, Cairo, Egypt; 2 Psychiatric and mental health nursing, faculty of nursing, Alexandria university, Alexandria, Egypt; 3 Nursing administration, faculty of nursing, Aswan university, Aswan, Egypt

**Keywords:** Organizational dehumanization, Work engagement, Nurses, Work stress

## Abstract

**Background:**

Organizational dehumanization has detrimental consequences for nurses’ wellbeing and leads to a stressful work environment. Moreover, it is very destructive to work engagement.

**Aim:**

To examine the mediating role of nurses’ work stress between organizational dehumanization and work engagement.

**Method:**

A cross-sectional research design was conducted with 245 staff nurses over a one-month period. The researchers used structured equation modeling.

**Results:**

Work engagement and organizational dehumanization levels were both moderate. In addition, the degree of job stress among the nurses was moderate, too. The results of the structural equation modeling showed that the association between organizational dehumanization and job engagement is partially mediated by work stress.

**Conclusions:**

For staff nurses to exhibit high levels of caring behaviors, this study emphasized the need to establish a work environment that employs tactics to improve workplace engagement and happiness. In addition to changing the organizational culture of nurses to eradicate organizational dehumanization and pressures related to the job.

**Supplementary Information:**

The online version contains supplementary material available at 10.1186/s12912-024-01841-z.

## Introduction

The need for quality healthcare is growing due to the aging of the population and the rapid development of health care, which presents significant obstacles for nurses in their profession. Nonetheless, the World Health Organization projects that there will be a global shortage of 5.7 million nurses by 2030 [1]. Despite the severe shortage (19.3 nurses per 10,000 populations) in Egypt, which is worse than the worldwide average, the issue continues to worsen [2]. The orientation of new nurses is projected to cost almost 1.3 times the yearly salary of a nurse, making nurse turnover costly. Additionally, it has an indirect effect whereby a shortage of nurses harms nurse satisfaction, productivity, patient safety, and nursing care quality [3]. These adverse effects led researchers and practitioners to search for years about what causes nurses to leave the workforce, but unfortunately, they have not yet entirely found solutions to effectively retain nurses [4].

Work engagement is a positive and rewarding sense of well-being while fulfilling one’s duty in the practice environment. Work engagement has three significant dimensions: vigor, dedication, and absorption [5]. Vigor refers to the willingness to invest effort in one’s work; dedication is related to participation, and absorption is related to concentration and absorption in one’s work [6].

Previous research has demonstrated that work engagement can raise the standard of nursing care by encouraging nurses to use all of their skills and knowledge [7]. High-job-engagement nurses demonstrated more compassionate behavior, job satisfaction, and employee efficiency [8]. Low burnout and less desire to leave the profession are also linked to nurse engagement [9]. Therefore, assessing, maintaining, and promoting worker engagement has become essential for many organizations [10]. There is a strong correlation between perceived organizational support and numerous favorable outcomes. Take the subjective well-being, performance, and work engagement study about perceived organizational support [11, 12].

In contrast, “organizational dehumanization” refers to how workers feel mistreated by the company because of their interactions, which often involves treating them like robots. Instead of treating people as human beings, showing less regard for their dignity, and using them as a tool to further organizational goals while showing less willingness and empathy [13, 14]. They will perform less well and have more attitudes of not being fully human. Additionally, workers who feel dehumanized by their employers are more likely to show signs of stress and dissatisfaction with their professions [13].

## Relationship between organizational dehumanization and work engagement

Organizational dehumanization is a concept that has lately come to light as being harmful to both individuals and organizations. Studies from different sectors have demonstrated that working in a dehumanized environment negatively affects both people and the organization, whose expenses are frequently considerably higher than others. Experiences of dehumanization at work are associated with adverse employment outcomes for certain people, including tardiness, work sabotage, organizational theft, absenteeism, and divulging trade secrets, all of which are unproductive work behaviors [15, 16]. Moreover, dehumanization will lead to counterproductive work behaviors such as alienation, high burnout, depression, and mental health symptoms [17]. Moreover, at the organizational level, working in a dehumanized organization often leads to quality issues, poor work performance, and poor quality within the organization, decreasing their level of working engagement [18].

Further, this concept is investigated with psychometric strain, job satisfaction, emotional exhaustion, and turnover intention [19]. However, there may also be a strong correlation between it and work engagement because employees’ opinions of the organization influence their willingness to participate at work. Moreover [20], recommended investigating it with other outcomes, such as work engagement. Thus, consistent with the recent research suggestions, this research will cover a gap by investigating the outcomes of organizational dehumanization, specifically work engagement.

Interactions between two partners who engage as individuals and institutions instead of partnerships between separate parties [21]. Workers are expected to socially exchange relationships with their company [22]. Therefore, these relationships influence workers’ behavior. People precisely repay what they receive to balance the exchanges with partners with whom they have social exchange relationships [23, 24]. In line with the Social Exchange Theory, workers who experience a loss of humanity will perform less well and be engaged less in work. People are driven to retreat when they believe they are not getting fair access to socio-emotional resources [20].

## Organizational dehumanization and work stress

The National Institute for Occupational Safety and Health (NIOSH) describes work stress as “"the negative physical and emotional reactions that occur when the worker’s needs, resources, or talents do not match the requirements of the job” [24]. The following are some of the sources of stress that nurses face at work: stress brought on by the working environment (professional responsibilities overloading organizations, excessive work, working shifts, inadequate remuneration that is not commensurate with the effort put in, and exposure to elements that may be detrimental to one’s bodily or mental well-being), stress regarding relationships with coworkers (the therapy team’steam’s milieu, interpersonal dynamics, and disputes between nurses and other team members) [25].

Dhanani, and LaPalme, (2019) argued that employee mistreatment fosters work stress. People’s psychological systems are impacted by such stressful interactions with organizations, which negatively alter their perspectives and reduce their resources [26]. Moreover, Cray (2010) found that there is a positive relationship between dehumanization and, individual emotional resources and work stress [27].

Additionally, based on the Conservation of Resources theory, organizational management may lead employees to believe that one stressor leads to another through a spiral effect [29]. The idea that employees are prone to develop rude colored glasses as perceiving any organizational stressor increases their perceptions of deviant behavior can be used to explain why it intensifies their sensitivity to perceive further mistreatment when employees are exposed to mistreatment instead from people or organizational point of view [26]. The implication is that if organizational dehumanization exists, it could make people feel under stress at work [29].

The lack of data on how dehumanization and job stress affect nursing professionals’ work engagement is troubling, given the state of the nursing workforce today. In light of the enormous need for highly skilled nurses and the steep expense of finding a replacement for an experienced nurse, it is more important than ever to look at how this demanding workplace affects nurses’ commitment to their jobs. This study was carried out to investigate the connection between organizational dehumanization and job engagement and assess the possible direct and moderating impacts of work stress in Egypt. Therefore, this study was created to fill the information vacuum in this area, strengthening healthcare professionals’ responses and enabling organizations to deliver reliable treatment. We also hope that by shedding light on the complex webs of organizational dehumanization and job stress, the nursing staff will never again be exposed to such actions. Therefore, the study aimed to investigate how nurses nurses work stress mediated the relationship between organizational dehumanization and job engagement.

## Research questions

- What are the levels of organizational dehumanization, work engagement, and work stress among staff nurses?
- Is there a relationship between organizational dehumanization, work engagement, and work stress among staff nurses?
- Is there a mediating effect for work stress between organizational dehumanization and work engagement among staff nurses?

## Materials and methods

### Research design and setting

This cross-sectional correlational descriptive study was conducted at two hospitals affiliated with Ain Shams University Hospitals. These Hospitals, namely Ain Shams University Hospital and Cardiovascular Hospital. Ain Shams University Hospital is a 606-bed teaching hospital. It is one of the biggest hospitals in Egypt and the Middle East, with a wide catchment area sub-serving a vast population. It is also a tertiary referral Centre for many hospitals nationwide and commonly from other nearby countries.

## Participants and sample size calculation

**Fig. 1:**
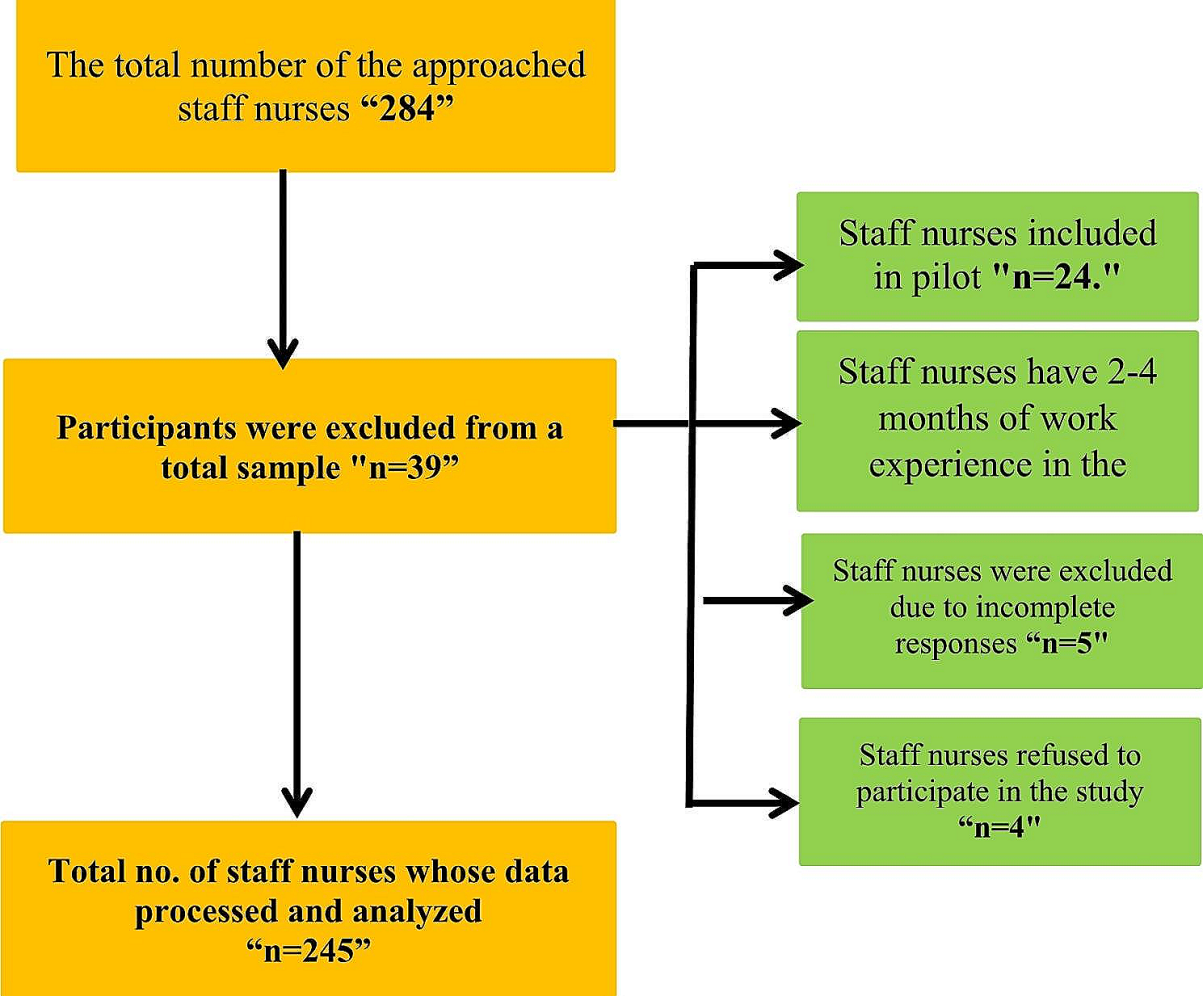
Participants’ recruitment process flow chart

Four hundred nurses comprised the entire population for the sample size calculation, with a 5% acceptable error, 50% anticipated frequency, and a 97% confidence coefficient. According to the Epi-Info-7 Programme, the bare minimum sample size was 216. Two hundred forty-five nurses out of the target population for recruitment in this research answered and filled out the questionnaire. However, 5 nurses were excluded due to incomplete responses, 6 staff nurses have 2–4 months of work experience in the same unit in which they were currently working, and 4 Staff nurses refused to participate in the study (Fig. 1). All recruited staff nurses must meet the following inclusion criteria: (a) have a certified licensed staff nurses and (b) have at least 6 months of work experience in the same unit in which they were currently working. The nurses who were in training or temporarily on leave throughout the data collection period were excluded from the study.

## Measurements

Four-measures were used for data collection.

**Tool 1. Socio-demographics**: Age, marital status, the number of children they have, where they live, educational background, department, and academic rank, as well as sex, were all included.

**Tool 2. Organizational dehumanization scale.** This scale consists of 11- items and developed by **Caesens et al. (2017)** [30]. Nurses were asked to rate statements such as ‘My organization makes me feel that one worker is easily as good as any other.’ The scoring system of organizational dehumanization was a five-point Likert scales ranging from strongly agree= [5] to strongly disagree= [1]. Items were scored 5, 4, 3, 2, and 1 for the responses “strongly agree,” “agree,” “neutral,” “disagree,” and “strongly disagree,” respectively. The researchers obtained the average score for each component, and the overall scale score (α = 0.90) high score reflects a high level of nurses’ dehumanization. In this study, the Cronbach α value was determined as 0.85.

**Tool 3. Work Engagement scale**. We used the nine-item Utrecht Work Engagement Scale to measure work engagement [31]. They responded to nine statements on a seven-point Likert scale anchored by “1” = strongly disagree to “7” agree. This scale is valid and has high internal consistency (Cronbach’s alpha = 0.91) as previously reported, while in the current study, the average score for each component was obtained by the researchers, and the overall scale score (α = 0.93) higher scores implied a higher level of nurses’ work engagement.

**Tool 4. Work stress scale**. The scale was created by De Bruin (2006) and has nine items on a 5-point Likert scale [32]. High scores imply a high level of job stress, whereas low scores denote a low level of work stress. According to Teles’ validity and reliability analysis of the scale in Turkey, the scale’s Cronbach alpha score is 0.91 [33]. The Cronbach alpha value for this study was found to be 0.92.

## Tools validity

The three instruments were modified, translated into Arabic, and then back into English. Four professors and two lecturers from the Nursing Administration Department were among the six experts who received the tools to examine, evaluate, and remark on content validity, question types, and item clarity. Their suggestions were considered to ensure accuracy and prevent the study from being jeopardized. A confirmatory factor analysis was carried out for organizational dehumanization, general work stress scale, and job engagement to assure accuracy. The Kaiser-Meyer-Olkin (KMO) and Bartlett Test of Sphericity were first used to evaluate sample adequacy. A Bartlett Test of Sphericity significance level of 0.05 is required, with a minimum KMO value of 0.60. The results revealed a value of 0.899 (P 0.000) for the organizational dehumanization scale, a value of 0.918 (P 0.000) for the general job stress scale, and a value of 0.931 (P 0.000) for the work engagement scale. Factor loadings for each construct examined in this investigation exceeded the suggested level of 0.70 [34], supporting the construct validity of the scales. Additionally, all research variable dimensions’ average variance extracted (AVE) values above 0.50 demonstrate that convergent validity is fulfilled [35] (**Supplementary file**).

## Ethical consideration

The study’s settings to gather the necessary data were authorized by the research ethics committee (REC) of the faculty of nursing at Ain-Shams University. The participating staff nurses were informed of the research’s objectives and given the freedom to deny participation or withdraw at any point before finishing the study instruments without facing any consequences. Nursing staff members who consented to participate in the study submitted signed, written, informed consent electronically. The confidentiality of the study’s data was ensured.

## Pilot study and reliability

The purpose of the pilot study, which involved 10% of the staff nurses (*n* = 24) from the context above, was to evaluate the practicality and clarity of the items, identify any potential difficulties or issues that might arise during data collection, and measure the time needed to complete the tools. The internal consistency of the items was assessed using Cronbach’s alpha coefficient test to assess the dependability of research tools.

## Data collection

The study was carried out with official approval from the Ain Shams University nursing faculty. Via a free online survey tool called Google Forms, data were gathered via a web-based survey. Nursing students were engaged in distributing the survey forms over WhatsApp Platforms because of their continuing attendance at the hospital for training and education, and they did so after distributing the survey link and invitation to nurses. Data was collected for one month, from July 15, 2023, to August 15, 2023. An online informed consent form was created, and participants had to agree to it by clicking an “I agree” button before moving on to the survey questions. Every survey question was made mandatory, meaning that partial responses could not be submitted. The researchers closed the survey page once the replies reached the estimated sample size.

### Statistical analysis

IBM SPSS Statistics (Version 23) and IBM SPSS AMOS (Version 23) were used to analyze the data. Frequency and percentage descriptions of the participant’s demographic characteristics were used. Means and standard deviations were used to define the three primary study variables (organizational dehumanization, work engagement, and nurses’ work stress). To find variations in the research variable based on demographic characteristics, an independent sample t-test and one-way analysis of variance were utilized. The correlation between the vital research variables was ascertained using Pearson’s correlation analysis. Regression analyses were used to calculate the direct effect of organizational dehumanization on work engagement. The indirect effect of organizational dehumanization on work engagement among nurses through nurses’ work stress was investigated using a structural equation model. To confirm the validity of the scale items utilized in this study, Cronbach’s alpha and composite reliability (CR) were computed. Additionally, several confirmatory factor analyses were carried out to guarantee the accuracy of the study constructs.

## Results

Table 1 shows that most subjects were 30 or younger (75.9%), and more than half of the studied nurses were female (60%). Approximately half of the studied subjects were single (49%), and the other half were married (50.2%). About two-thirds of the 66.9% of the studied nurses have a diploma from a technical health institute or nursing division, and about 40.2% have less than 5 years of experience since graduation. In terms of years of experience in their current job, more than half of the studied subjects have less than 5 years of experience.

**Table 1:** Participants’ the socio demographic of staff nurses (n = 245)

Characteristic	Category	*n.* (%)
**Age**	30 years or less	186(75.9)
	31–40 years	51(20.8)
	More than 40	8(3.3)
**Sex**	Male	98(40.0)
	Female	147(60.0)
**Marital status**	single	120(49.0)
	married	123(50.2)
	divorced	2(0.8)
**Academic Qualification**	PH.D	1(0.4)
	Diploma of Technical High School of Nursing	25(10.2)
	Diploma of the Technical Health Institute, Nursing Division.	164(66.9)
	Bachelor of Nursing	25(10.2)
	Higher diploma after bachelor’s degree	4(1.6)
	Other qualifications	26(10.6)
**Number of years of experience since graduation**	Less than 5 years	101(41.2)
	5 - Less than 10 years	84(34.3)
	10–15 years	29(11.8)
	More than 15 years	31(12.7)
**Number of years of experience in your current job**	Less than 5 years	130(53.1)
	5 - Less than 10 years	81(33.1)
	10–15 years	17(6.9)
	More than 15 years	17(6.9)

Table 2 show that half of the studied subjects in the present study reported a moderate degree of organizational dehumanization (49.39%), followed by a low degree (34.9%). The items “my organization makes me feel that my only importance is my performance at work” and “the only thing that counts for my organization is what I can contribute to it” were reported with high mean scores (3.07 ± 1.31 and 3.21 ± 133, respectively).

**Table 2:** Distribution of the study subjects according to Organizational dehumanization

Organizational dehumanization	(*n* = 245)				
	Mean ± SD	Mean score percent	Level	Frequency	percent
My organization makes me feel that one worker is easily as good as any other.	2.37 ± 1.15	47.35%	Low (11–25)	88	35.92%
My organization would not hesitate to replace me if it enabled the company to make more profit.	2.56 ± 1.20	51.27%			
If my job could be done by a machine or a robot, my organization would not hesitate to replace me by this new technology.	2.58 ± 1.28	51.59%			
My organization considers me as a tool to use for its own ends	2.50 ± 1.24	49.96%	Moderate (26–40)]	121	49.39%
My organization considers me as a tool devoted to its own success	2.87 ± 1.26	57.47%			
My organization makes me feel that my only importance is my performance at work	3.07 ± 1.31	61.31%			
My organization is only interested in me when it needs me	2.78 ± 1.27	55.51%	High (41–55)	36	14.69%
The only thing that counts for my organization is what I can contribute to it were	3.21 ± 1.33	64.16%			
My organization treats me as if I a robot	2.64 ± 1.31	52.82%			
My organization considers me as a number	2.54 ± 1.19	50.86%			
My organization treats me as if I were an object	2.43 ± 1.22	48.65%			

Table 3 shows that half of the studied subjects in the current study reported low to moderate degrees of general work stress (50.20% and 37.14%, respectively). “Does work make you so stressed that you wish you had a different job?” And “Do you worry about having to wake up and go to work in the morning?” reported the high mean scores of 2.60 ± 1.24 and 2.50 ± 1.29, respectively.

**Table 3:** Distribution of the study subjects according to General Work Stress Scale

General Work Stress Scale	(*n* = 245)				
	Mean ± SD	Mean score percent	Level	Frequency	percent
Does work make you so stressed that you wish you had a different job?	2.60 ± 1.24	52.00%	Low (19–20)	123	50.20%
Do you get so stressed at work that you want to quit?	2.49 ± 1.22	49.71%			
Do you worry about having to wake up and go to work in the morning?	2.50 ± 1.29	50.04%			
Do you find it difficult to sleep at night because you worry about your work?	2.43 ± 1.29	48.65%	Moderate (21–32)	91	37.14%
Do you get so stressed at work that you forget to do important tasks?	2.11 ± 1.19	42.20%			
Does work make you so stressed that you find it hard to concentrate on your tasks?	2.09 ± 1.19	41.88%			
Do you spend a lot of time worrying about your work?	2.21 ± 1.21	44.24%	High (33–45)	31	12.65%
Do you feel like you cannot cope with your work anymore?	2.15 ± 1.25	42.94%			
Does work make you so stressed that you lose your temper?	2.48 ± 1.33	49.55%			

Table 4 shows that more than half of the studied subjects in the present study reported high general work engagement (56.33%). The items “I am enthusiastic about my job” and “I found the work that I do full of meaning and purpose” reported high mean scores of 4.18 ± 2.03 and 4.05 ± 2.12, respectively.

**Table 4:** Distribution of the study subjects according to work engagement

Work engagement	(*n* = 245)				
	Mean ± SD	Mean score percent	Level	Frequency	percent
At my work, I feel bursting with energy	3.07 ± 1.85	51.22%	Low (0–17)	40	16.33%
At my job, I feel strong and vigorous	3.67 ± 2.01	61.09%			
I am enthusiastic about my job	4.18 ± 2.03	69.59%			
My job inspires me	3.96 ± 1.99	65.92%	Moderate (18–35)	67	27.35%
When I get up in the morning, I feel like going to work	3.57 ± 2.14	59.46%			
I am immersed in my work	3.98 ± 2.04	66.26%			
When I am working, I forget everything else around me	4.01 ± 2.00	66.80%	High (36–54)	138	56.33%
I feel happy when I am working intensely	3.72 ± 2.11	62.04%			
I find the work that I do full of meaning and purpose.	4.05 ± 2.12	67.55%			

Table 5 shows the correlation matrix between organizational dehumanization, general work stress, and work engagement among nurses. The organizational nurse’s dehumanization was positively and significantly correlated with work stress (*r* = 0.387, *P* < 0.01) and insignificantly negatively correlated with work engagement (*r* = -0.041). The table also shows that general work stress negatively correlates with work engagement (*r* = -0.225, *P* < 0.01).

**Table 5:** Correlations between the study variables (*n* = 245)

Variable	General Work Stress Scale	Work engagement
**Organizational dehumanization**	0.387**	-0.041
**General Work Stress Scale**		-0.225**
**Work engagement**		

**Fig. 2:**
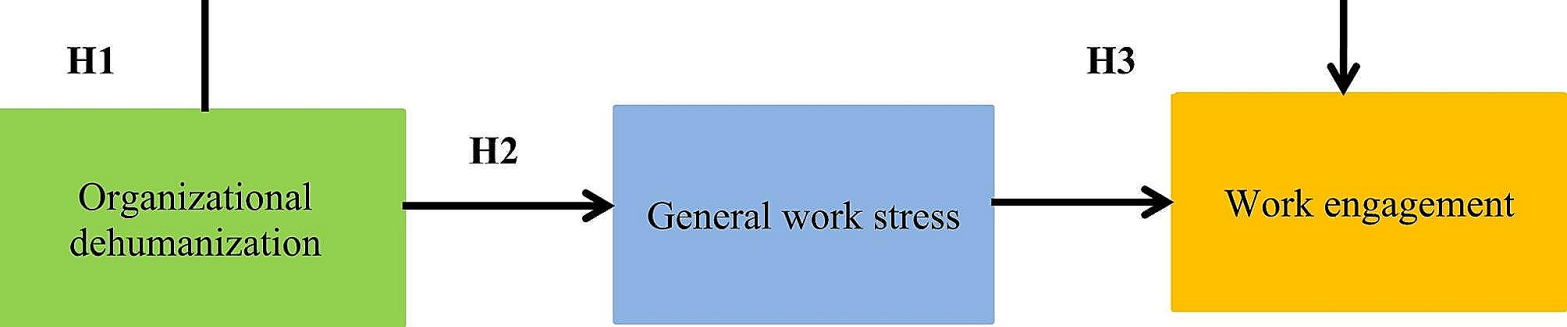
Conceptual model

According to the study’s authors, to test the study hypotheses, the effect of organizational dehumanization as an independent variable on nurses’ job engagement as an outcome variable, removing the influence of nurses’ general work stress, was tested using regression analysis to determine the direct effect. As well as using a structural equation model, the indirect effect (the impact of organizational dehumanization of nurses as an independent variable on nurses’ job engagement as an outcome variable via general work stress as a mediating variable) is examined (Fig. 2).

**Fig. 3:**
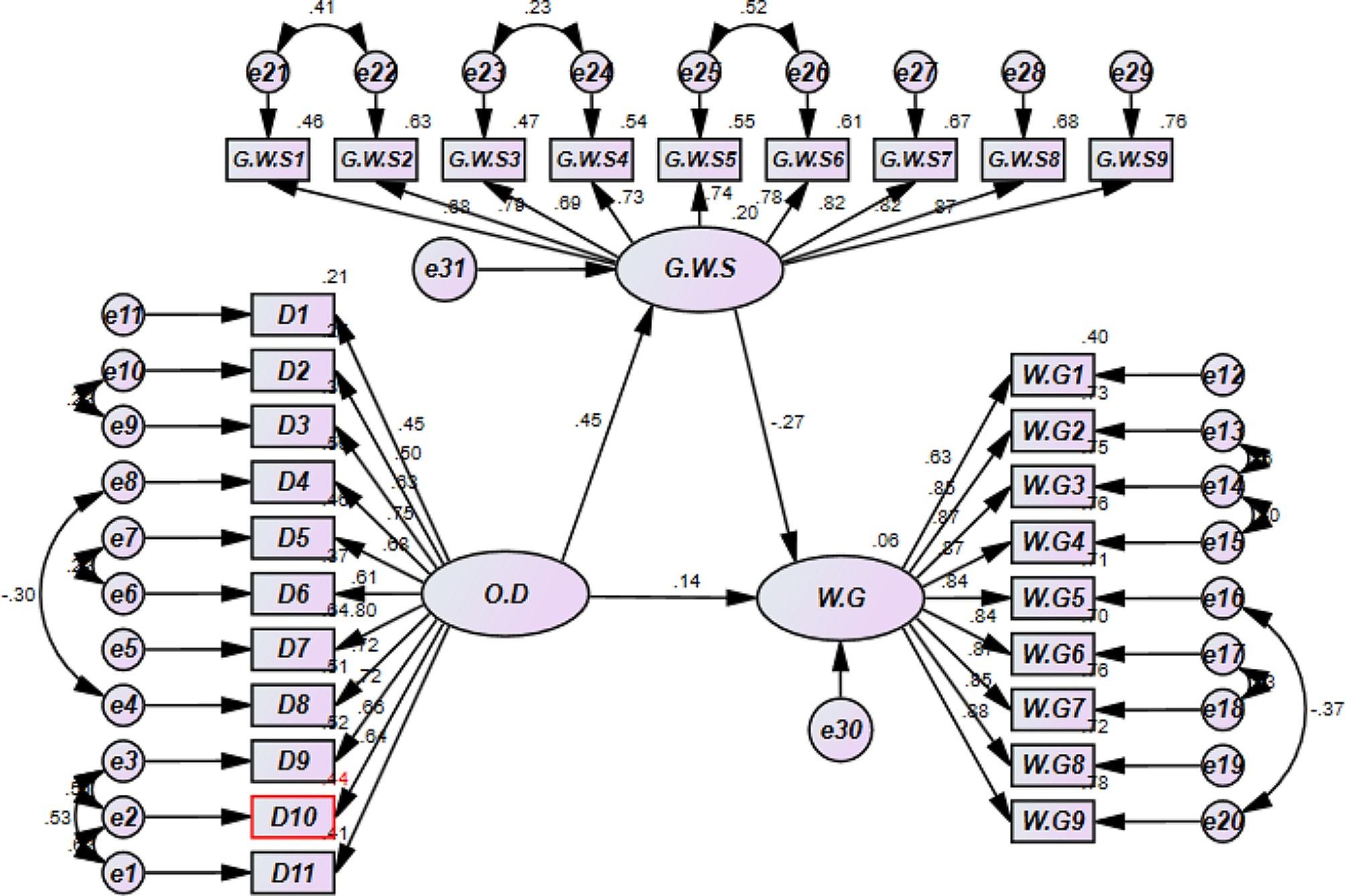
Mediation model Model fit summery X^2^=663.333, df=361, X^2^/df=1.837, CFI=0.943, TLI=0.936, RMSEA=0.059Model abbreviation (OD: Organizational Dehumanization, GWS: General work stress, WG: Work engagement

Figure 3; Table 6 results showed that organizational dehumanization of nurses was positively correlated with general work stress among nurses (*r* = 0.446, p 0.001), supporting hypothesis H2 and that general work stress among nurses was negatively correlated with work engagement (*r* = -0.267, p 0.001). Additionally, the relationship between organizational dehumanization and job engagement was no longer significant, and the beta-coefficient was reduced (*r* = 0.137, *P* = 0.082) once general work stress was included in the model. The bootstrapping test revealed that the indirect effect of organizational dehumanization of nurses on nurses’ work engagement through general work stress did not include zero (-0.119), indicating that the indirect effect was significant. The test used 5,000 bootstrapping resamples. Together, these findings indicated that nurses’ general work stress mediates the relationship between nurse organizational dehumanization and nurses’ work engagement. Furthermore, Fig. 1 shows the model’s satisfactory fit (X2 = 663.333, df = 361, X2/df = 1.837, CFI = 0.943, TLI = 0.936, RMSEA = 0.059).

**Table 6:** Mediation effect of nurses’ work stress between nurse Organizational Dehumanization and nurses’ work engagement (*n* = 245)

	Standardized Regression Weights	Estimate	S.E.	C.R.	*P*
**Organizational Dehumanization to General work stress**	0.446	0.474	0.085	5.602	0.000
**General work stress to Work engagement**	− 0.267	− 0.376	0.114	-3.303	0.000
**Direct effect Organizational Dehumanization to Work engagement**	0.137	0.204	0.117	1.740	0.082
**Indirect effect of Organizational Dehumanization to Work engagement**	-0.119				
**Total effect**	0.017				0.000

Estimate based on 5,000 bootstrap re-samples

## Discussion

Employees’ psychological suffering is made worse by work-related stress [36]. Due to the intense stress caused by their demanding schedules, the extent of their tasks, and interpersonal problems, hospital nurses have an abnormally high frequency of substantial psychological discomfort. When nurses suffer psychological distress, their work turnover and administrative leave rates increase, which may cause medical mistakes and problems with patient interaction [37]. Among healthcare professionals, nurses face the most significant levels of occupational stress and are particularly vulnerable to various stressors and workplace expectations that may harm their level of involvement at work [38]. This study aimed to determine if nurses’ work stress mediated the link between organizational dehumanization and job engagement.

First, our results showed that half of the studied subjects in the current study reported moderate-to-high degrees of general work stress. From the researcher’s point of view, it may be related to the low experience among the studied nurses, as most were aged less than 30 years and had less than five years of experience in nursing work. In this respect, Diab & Elnagar, (2019) concluded that the more youthful the nurse is, the more stress she experiences [39]. In addition, psychological or physical abuse, an extremely demanding job, a lack of staff members, a high patient frequency, the ensuing excessive burden, and infection exposure are additional causes that frequently cause nurses to stress [40]. Similarly, Arbabisarjou et al., (2017) and Seada (2017) reported that more than three-quarters of the studied nurses had a moderate stress level in their work [38, 41].

Work stress has detrimental effects on the organization, including job dissatisfaction, lower performance, and increased organizational dehumanization [42]. Second, the current results revealed a moderate degree of organizational dehumanization. This may be attributed to organizational workers’ mistreatment by placing undue pressure on them through heavy workloads and mechanical structures while ignoring the humanistic perspective. Consistent with the current results, some previous researchers found that an organization can misuse, hinder, damage, or otherwise negatively affect its employees [43]. The most severe problem facing organizations nowadays is the negative aspects of employee behaviors due to depleting employees’ psychological resources [44]. When employees perceive their relationship with the organization as disruptive and mistreated by the organization, it leads to undesirable organizational outcomes [45].

Sarwar (2020) also offers practical tips for managers, like promoting civility, respect, and workplace involvement [46]. Three major factors that are combining to make work engagement in nursing strategically important are a global shortage of nurses, who make up the largest group of healthcare providers, political, will contain the growth of rising healthcare costs in industrialized countries and a medical error rate that jeopardizes national health [47]. Employee engagement is one of the most influential metrics for gauging an organization’s progress toward its goals, vision, and core values [48]. Fortunately, the current study results showed that more than half of the studied subjects reported high general work engagement. This could be related to the nurses’ ability to adapt to changes in their work environment. In addition, as most of the studied nurses are married, the work may be considered the primary source of their income, so they are obliged to be involved. Additionally, Zamaniniya et al. (2021) reported that the humanistic approach to patient care enhances a nurse’s sense of purpose and engagement at work [49].

Consistent with the current results, Egyptian research studies found that the studied nurses are highly engaged in their work [50]. On the contrary, other studies showed that nurses generally had low levels of work engagement, which was linked to adverse outcomes such as a higher turnover rate, low job satisfaction, and subpar performance of job duties [51].

Third, the findings proved that general work stress was positively associated with nurses’ organizational dehumanization and negatively associated with their work engagement. This reciprocal relationship is interpreted by the fact that when employees believe their organization treats them like robots and does not care about their interests; it could lead to work stress and burnout [52]. Supporting the current results, Rubbab et al., (2022) reported that employees usually perceive physical and psychological strain as a reaction to organizational mistreatment through perceived dehumanization [53]. Also, prior research discovered a significant negative association between the degree of job engagement and work pressure, indicating the need for mitigating techniques to provide a more effective hospital organization [54].

The study’s final hypothesis was that the association between organizational dehumanization and nurses’ job engagement was mediated by work stress. According to this hypothesis, we discovered that the association between organizational dehumanization and nurses’ job engagement was nearly to be fully mediated by work stress. This finding showed that nurses’ work distress rises when the organization dehumanizes its nursing personnel, negatively influencing their work engagement and vice versa [53]. The importance of a thriving organizational environment and practices is the first time in nursing that contextual solutions and information are offered to organizations and managers. Lowering workplace stress and illuminating the adverse effects of organizational dehumanization eventually improves organizational performance.

## Limitations and implications for Future Research

This study has a few limitations. First, convenience sampling was used, which may have restricted the generalizability of these findings. Future studies are encouraged to use random samples. Second, the study’s cross-sectional design did not establish causal links between the study variables. It is advised to do further longitudinal studies to corroborate these associations. Finally, Future research should concentrate on specific tactics or cures for organizational dehumanization and nurses’ work stress. Furthermore, figuring out the causes of dehumanization and work-stress attitudes and offering guidance on managing and controlling them will be of more significant concern to researchers and practitioners on a larger scale.

## Conclusions

According to this study, organizational dehumanization, work stress, and their effects on Egyptian nurses’ work engagement were all examined. The organizational nurse’s dehumanization was positively and significantly correlated with work stress and insignificantly negatively correlated with work engagement; likewise, general work stress negatively correlates with work engagement. Healthcare organizations should implement strategies to increase job engagement and satisfaction among nurses so that they engage in high levels of caring behaviors.

## Implications for nursing management

Dehumanization is an undesirable concept that lessens originality. As a result, people may decide to quit supporting an organization. As a result, managers must design interventions to teach staff members that they are valued as individuals rather than expendable goods. To make staff members feel less dehumanized and more supported, hospitals and their managers may apply particular Human Resources practices, such as lowering workload, enhancing job stability, and providing training and development opportunities for their growth and grooming. Workshops, conferences, and team-building exercises involving active engagement between individuals from various levels of management are far better at reducing employees’ sense of dehumanization. Organizations must understand that treating employees as human beings comes before considering their performance. Moreover, the study results highlight the need for establishing a work environment that promotes job engagement through inspirational leadership, strong mentoring relationships between experienced and novice nurses, treating nurses fairly, better communication, nurse support, encouraging a supportive workplace, and rewards and incentives.

### Electronic supplementary material

Below is the link to the electronic supplementary material.

Supplementary Material 1

## Data Availability

The datasets used and/or analyzed during the current study available from the corresponding author on reasonable request.
