# Supplementary material for: The relationship between organizational dehumanization and work engagement: the mediating effect of nurses’ work stress
Source: BMC Nurs. 2024 Mar 22;23:193. doi: 10.1186/s12912-024-01841-z (PMC10958847; doi:10.1186/s12912-024-01841-z)
Supplement: Supplementary file 1 — Supplementary Material 1 [file 12912_2024_1841_MOESM1_ESM.docx]

**Factor analysis of organizational dehumanization, general work stress scale, and work engagement**

**Exploratory** **Factor Analysis of** Organizational dehumanization

| Component | Extraction | Extraction Sums of Squared Loadings | | Component Matrix of Resilience questions | |
| --- | --- | --- | --- | --- | --- |
|  |  | **Total** | **% of Variance** | **1** | **2** |
| Q1 | 0.409 | 5.484 | 49.857 | 0.503 | 0.395 |
| Q2 | 0.581 | 1.146 | 10.421 | 0.543 | 0.535 |
| Q3 | 0.529 |  |  | 0.688 | ــــــــ |
| Q4 | 0.577 |  |  | 0.758 | ــــــــ |
| Q5 | 0.525 |  |  | 0.697 | ــــــــ |
| Q6 | 0.439 |  |  | 0.657 | ــــــــ |
| Q7 | 0.652 |  |  | 0.808 | ــــــــ |
| Q8 | 0.505 |  |  | 0.685 | ــــــــ |
| Q9 | 0.820 |  |  | 0.809 | -0.408 |
| Q10 | 0.805 |  |  | 0.774 | -0.453 |
| Q11 | 0.788 |  |  | 0.772 | -0.437 |
| Kaiser-Meyer-Olkin Measure of Sampling Adequacy.( KMO) | 0.899 | | | | |
| Sig. | 0.000 | | | | |
| α | 0.897 | | | | |

CR, Composite Reliability. AVE, Average Variance Extracted.

**Exploratory** **Factor Analysis of General Work Stress Scale**

| Component | Extraction | Extraction Sums of Squared Loadings | | Component Matrix of Resilience questions |
| --- | --- | --- | --- | --- |
|  |  | **Total** | **% of Variance** |  |
| Q1 | 0.530 | 5.870 | 65.222 | 0.728 |
| Q2 | 0.682 |  |  | 0.826 |
| Q3 | 0.571 |  |  | 0.756 |
| Q4 | 0.607 |  |  | 0.779 |
| Q5 | 0.642 |  |  | 0.801 |
| Q6 | 0.698 |  |  | 0.835 |
| Q7 | 0.700 |  |  | 0.837 |
| Q8 | 0.681 |  |  | 0.825 |
| Q9 | 0.759 |  |  | 0.871 |
| Kaiser-Meyer-Olkin Measure of Sampling Adequacy.( KMO) | 0.918 | | | |
| Sig. | 0.000 | | | |
| α | 0.933 | | | |

CR, Composite Reliability. AVE, Average Variance Extracted.

**Exploratory** **Factor Analysis of** Work engagement

| Component | Extraction | Extraction Sums of Squared Loadings | | Component Matrix of Resilience questions |
| --- | --- | --- | --- | --- |
|  |  | **Total** | **% of Variance** |  |
| Q1 | 0.481 | 6.651 | 73.898 | 0.693 |
| Q2 | 0.777 |  |  | 0.882 |
| Q3 | 0.823 |  |  | 0.907 |
| Q4 | 0.808 |  |  | 0.899 |
| Q5 | 0.715 |  |  | 0.846 |
| Q6 | 0.756 |  |  | 0.869 |
| Q7 | 0.801 |  |  | 0.895 |
| Q8 | 0.731 |  |  | 0.855 |
| Q9 | 0.759 |  |  | 0.871 |
| Kaiser-Meyer-Olkin Measure of Sampling Adequacy.( KMO) | 0.931 | | | |
| Sig. | 0.000 | | | |
| α | 0.955 | | | |

CR, Composite Reliability. AVE, Average Variance Extracted.
